# Impact of Sapphire Substrate Reconstruction on the Structural, Electronic, and Photonic Properties of MoS_2_

**DOI:** 10.1002/smll.202511179

**Published:** 2026-03-11

**Authors:** Riccardo Torsi, Kyle T. Munson, Daniel Eppler, Jo Laura, Furkan Turker, Maxwell A. Feidler, Joerg Appenzeller, Yu‐Chuan Lin, Joshua A. Robinson

**Affiliations:** ^1^ Department of Materials Science and Engineering The Pennsylvania State University University Park Pennsylvania USA; ^2^ Quantum Measurement Division National Institute of Standards and Technology Gaithersburg Maryland USA; ^3^ Department of Electrical and Computer Engineering Purdue University West Lafayette Indiana USA; ^4^ Department of Chemistry The Pennsylvania State University University Park Pennsylvania USA; ^5^ Department of Materials Science and Engineering National Yang Ming Chiao Tung University Hsinchu City Taiwan; ^6^ Materials Research Institute The Pennsylvania State University University Park Pennsylvania USA; ^7^ Department of Physics The Pennsylvania State University University Park Pennsylvania USA

**Keywords:** 2D transition metal dichalcogenides, atomic force microscopy, charge transfer, chemical vapor deposition, MoS_2_, optical spectroscopy, sapphire surface reconstruction, substrate/film coupling

## Abstract

Transition metal dichalcogenides (TMDs) are commonly grown on single‐crystal sapphire via chemical vapor deposition. Understanding how TMD–sapphire interactions affect the structural and photonic properties of TMDs is critical for accurately interpreting optical characterization and distinguishing intrinsic material properties from substrate‐induced effects. Here, we report the impact of sapphire surface reconstruction on the structural and photonic properties of MoS_2_ monolayers grown by metal–organic chemical vapor deposition. Kelvin probe force microscopy measurements indicate that sapphire step‐edge formation during growth enhances surface potential variations, consistent with substrate‐induced charge doping at the MoS_2_/sapphire interface. Raman and photoluminescence measurements reveal that film/substrate interactions introduce considerable strain and substrate‐induced charge doping inhomogeneities across the film, broaden and shift MoS_2_ Raman modes, and induce heterogeneous emission. In contrast, photoluminescence, Raman, and transport measurements of MoS_2_ films transferred away from their sapphire growth substrates reveal uniform optical responses and device characteristics, indicating that the observed heterogeneity originates from interfacial coupling rather than intrinsic film quality. Together, these results demonstrate that growth‐induced TMD–substrate interactions not only influence optical metrology on as‐grown films but also impact the transfer processes that underpin device fabrication, emphasizing the importance of controlling interfacial effects for reliable characterization and integration of wafer‐scale 2D semiconductors.

## Graphical abstract

High‐temperature reconstruction of c‐plane sapphire during MOCVD growth of MoS_2_ fundamentally alters the optical and electronic properties of MoS_2_ epilayer by introducing step‐edge driven interfacial charge transfer. Raman, PL, and KPFM reveal non‐uniform doping and strain in as‐grown films, whereas transferred layers show uniform optical and electronic properties, which highlights the critical role of substrate reconstruction in TMD characterization and device performance.

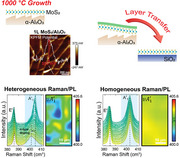

## 1 Introduction

2D transition metal dichalcogenides (TMDs) feature a unique combination of atomic‐scale thickness and remarkable (opto)electronic properties, rendering them leading candidates for next‐generation devices ranging from flexible photodetectors to ultra‐scaled field‐effect transistors (FETs) [1, 2, 3]. Achieving reliable, wafer‐scale production of TMDs is essential for their practical implementation [4]. Chemical vapor deposition (CVD), a process involving the reaction of precursor species in the vapor phase and their subsequent deposition onto a substrate, has emerged as the leading candidate to achieve this goal [5]. The efficacy of CVD in producing high‐quality TMD crystals hinges on the careful selection of growth substrate, which can impact the nucleation [6], orientation [7], and grain size [8] of the resultant TMD film [9]. Single crystalline sapphire is widely used to grow epitaxial TMD films because of its relative stability, ultra flat surface, and commensurate TMD and sapphire lattices [10]. Additionally, engineering the step‐and‐terrace morphology of sapphire substrates via various miscuts [6] and annealing procedures [11, 12], presents a promising avenue to manipulate TMD nucleation and growth mechanisms. For example, sapphire surface reconstruction can induce step‐edge nucleation and break the degeneracy of 0°/60° antiparallel domains, leading to films with unidirectionally aligned grain boundaries [6, 13, 14]. Furthermore, the pre‐growth annealing of sapphire substrates can aid unidirectional TMD epitaxy on sapphire by promoting uniform step‐bunching and surfaces with only one kind of atomic plane symmetry [15]. In CVD synthesis of TMDs on sapphire, high growth temperatures (> 800°C) are pivotal for achieving epitaxial growth and realizing high‐quality crystalline films [16]. Chubarov et al. showed that increasing the growth temperature from 850°C to 1000°C resulted in a dramatic narrowing of monolayer WS_2_ XRD φ‐scan peaks from 1.13° to as low as ≈ 0.09° [17]. Notably, the improvements in material quality achieved at high growth temperatures have also been demonstrated to directly result in superior FET device performance [18]. However, the high temperatures and aggressive chemical environments within CVD chambers can alter the sapphire's surface termination and reconstruct its morphology during TMD growth [19, 20, 21].

Understanding how substrate reconstruction influences the structural and photonic properties of TMDs is critical when using optical methods to characterize TMD properties and, subsequently, deconvolute these intrinsic material properties from effects arising from substrate topography. Raman and photoluminescence (PL) spectroscopy are two non‐destructive techniques widely used to assess the crystalline quality and uniformity of 2D TMDs [22, 23, 24, 25]. For example, investigators use Raman spectroscopy to determine TMD film thicknesses because the spectral separation between the $E_{2g}$ and $A_{1g}$ modes of TMDs, such as MoS_2_, are sensitive to the TMD layer number [26, 27], with the frequency gap widening in correlation to the sample thickness. The full width at half maximum (FWHM) of MoS_2_ Raman‐active modes is also used to determine sample quality, with broader peaks attributed to a higher degree of crystalline disorder [25]. Likewise, PL emission intensity and FWHM are routinely cited as indicators of TMD sample quality [22, 28].

However, despite the widespread use of Raman and PL spectroscopy to assess TMDs, strain and charge doping effects from the underlying substrate can shift the center frequencies of TMD Raman active modes [29, 30], and quench PL due to trion formation [31]. Moreover, substrate‐induced interactions can also influence the mechanical robustness and chemical integrity of TMD and other 2D material films transferred away from their growth substrates, impacting device performance [21, 32]. Investigators have made considerable efforts to understand how TMD/substrate interfaces influence the structural and photonic properties of TMDs grown or transferred to substrates such as SiO_2_, sapphire, mica, and hBN [33, 34]. However, the impact of sapphire substrate reconstruction on the structural and photonic properties of TMDs remains an open area of research.

In this work, we demonstrate that c‐plane sapphire (α‐Al_2_O_3_) reconstruction during metal‐organic chemical vapor deposition (MOCVD) synthesis of MoS_2_ has a pronounced impact on MoS_2_–substrate interactions and photonic properties. Atomic force microscopy (AFM) measurements reveal that increasing the MOCVD growth temperature from 900 °C to 1000 °C enhances sapphire surface reconstruction and step bunching processes. Verified by Kelvin probe force microscopy (KPFM), we find that sapphire step‐edges produce local surface potential variations, consistent with substrate‐induced charge doping at the MoS_2_/sapphire interface. Raman and PL microscopy corroborate these measurements by revealing heterogeneous strain, charge doping, and emission across MoS_2_ films grown at 1000 °C due to sapphire step‐edge formation during growth. However, transferring MoS_2_ films from their growth substrates to device relevant SiO_2_/Si reduces these heterogeneous substrate‐induced charge doping effects. Transport measurements of back‐gated field‐effect transistors (BGFETs) made from transferred films verify our optical measurements and reveal uniform device characteristics across the sample area. In addition, X‐ray photoelectron spectroscopy (XPS) measurements reveal residual Mo and S species remaining on the sapphire surface following film transfer. AFM characterization indicates that these residues are concentrated at sapphire step edges, where film–substrate interactions are strongest. These results demonstrate that growth‐induced interfacial interactions not only modify optical and electronic properties but also directly influence TMD‐substrate interactions that affect film integrity and transfer behavior.

## 2 Results and Discussion

**FIGURE 1:**
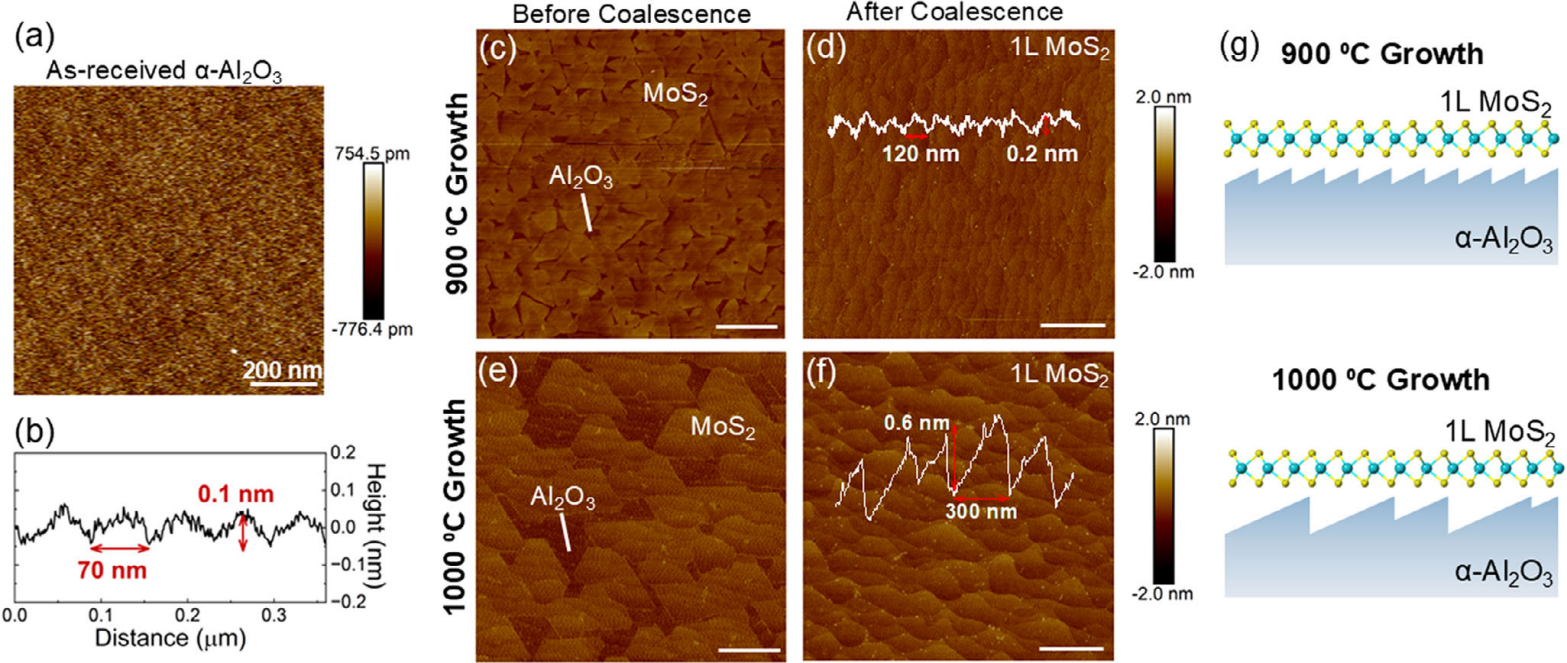
Influence of MoS_2_ growth temperature on sapphire (α‐Al_2_O_3_) substrate reconstruction. (a) Atomic force microscopy scans of an as‐received α‐Al_2_O_3_ substrate with corresponding line profile (b) showing the presence of step‐and‐terrace morphology. AFM scan of an MoS_2_ film grown on sapphire at 900°C, (c) before and (d) after monolayer coalescence. AFM scan of an MoS_2_ film grown on sapphire at 1000°C, (e) before and (f) after monolayer coalescence. In scans (c,e), individual domains can be resolved, showing larger, more uniformly aligned grains in the 1000°C sample compared to the 900°C analog. AFM scans of fully coalesced monolayer films in (d,f) show the modification of the step‐and‐terrace morphology of the underlying substrate highlighted by the overlying line profiles. Scale bars in (c‐f) are 400 nm. (g) Depiction of sapphire step edges after MoS_2_ growth at 900°C and 1000°C.

The morphology of monolayer MoS_2_ films grown on c‐plane sapphire substrates at different temperatures is initially examined (Figure 1). We synthesized MoS_2_ films in a custom‐built MOCVD reactor equipped with high‐purity Mo(CO)_6_ and H_2_S precursor sources using a multi‐step growth process as previously described [35]. This process produces coalesced monolayer films with < 1% bilayer coverage. We selected growth temperatures of 900°C and 1000°C to span the temperature range associated with the onset of pronounced sapphire substrate reconstruction, allowing us to directly probe its impact on film growth [36, 37]. A representative atomic force microscopy (AFM) scan of a coalesced monolayer film grown at 900°C is shown in Figure 1d. The underlying morphology originates from the step‐and‐terrace structure of the underlying sapphire substrate (miscut 0.2° ± 0.1° toward *m* axis $\left\langle 10\bar{1}0\right\rangle$). The as‐received sapphire substrate exhibits parallel steps oriented to the $\left\langle 11\bar{2}0\right\rangle$ direction (*a* axis), averaging 0.1 nm in height and 60 nm in width (Figure 1a,b).

At 900°C, the sapphire substrate is chemically stable, and we observe only minor modifications to the surface step‐and‐terrace structure. The surface roughness of the films is comparable to that of the starting substrate (*Ra =* 0.17 nm), and the average step height and terrace width increases to 0.20 nm (corresponding to c/6) and 100 nm, respectively (Figure 1d). Analysis of the monolayer film surfaces just prior to coalescence unveils the polycrystalline nature of the MoS_2_ films at 900°C, with triangular grains ranging in size from ≈ 100 nm to ≈ 300 nm. (Figure 1c). The range in grain sizes indicates that nucleation events are not limited to the early stages of growth but persist as initial grains grow laterally. The existence of diverse grain orientations results in the formation of grain boundaries during layer coalescence, which limit charge carrier transport and are generally unfavorable for (opto)electronic applications [38].

Elevating the growth temperature to 1000 °C enhances the crystalline quality of MoS_2_ films by simultaneously reducing MoS_2_ nucleation density and improving adatom diffusion on the sapphire surface [16]. Figure S1 shows the evolution of domain morphology in the early stages of growth for the two different temperatures. Increasing the growth temperature from 900 °C to 1000 °C reduces the nucleation density by more than a factor of two, accompanied and by increased adatom diffusion which leads to the growth of larger grains in the ripening stage. Examination of a sub‐coalesced monolayer film grown at 1000 °C reveals a noticeably different grain structure compared to the 900 °C analog (Figure 1e), with the 1000 °C film exhibiting increased grain sizes (≈500 nm) and a more uniform orientation, indicating improved epitaxial alignment with the underlying sapphire substrate. Additional statistical analysis of grain size and domain orientation comparing films grown at 900 °C and 1000 °C is provided in Figure S2. After increasing the growth time to achieve fully coalesced films, the AFM scan in Figure 1f reveals the step‐and‐terrace morphology of the underlying sapphire substrate. This assignment is confirmed by transferring the MoS_2_ films to SiO_2_/Si substrates that lack step and terrace morphology (Figure S3). Crucially, unlike the 900°C sample, the growth process significantly alters the step and terrace structure of the sapphire surface at 1000 °C (Figure 1g), causing the surface to exhibit a mixture of steps, some measuring 0.4 nm in height and ≈ 150 nm in width, and others 0.6 nm in height and ≈ 300 nm in width. Investigators observed similar surface modification in sapphire substrates annealed at high temperatures (>1000°C) and attributed it to the diffusion of atoms at the sapphire surface leading to step coalescence [19, 21, 36]. In addition to thermal effects, the chemical environment inside the MOCVD chamber can also alter sapphire surface chemistry and termination. For example, exposing sapphire substrates to a hydrogen chalcogenide precursor during high temperature MOCVD growth results in chalcogen termination of the sapphire surface [4, 13]. Therefore, we speculate that a combination of thermal effects and chalcogen surface termination is responsible for the sapphire surface reconstruction observed here.

**FIGURE 2:**
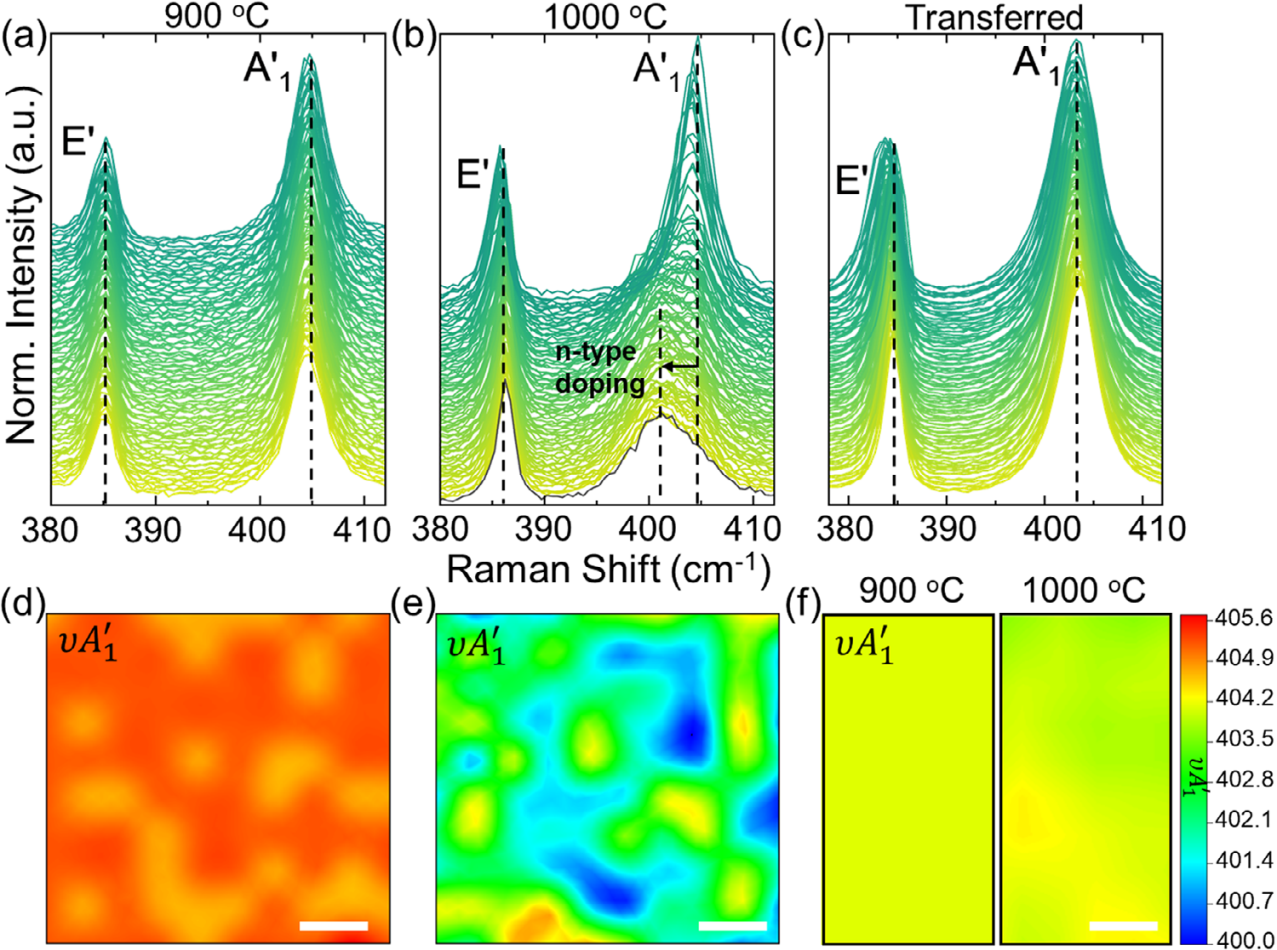
Influence of sapphire surface reconstruction on the structural properties of MoS_2_. Raman spectra collected from a 2500 µm^2^ mapped area for MoS_2_ films grown on α‐Al_2_O_3_ at (a) 900°C and (b) 1000°C. (c) Raman spectra of the 1000°C film after transfer to SiO_2_/Si. The in‐plane (*E*′) and out‐of‐plane ($A_{1}^{\prime }$) peaks are marked for each sample. The spectra are offset for clarity. Raman maps of $A_{1}^{\prime }$ peak position in cm^−1^ (υ$A_{1}^{\prime }$) for monolayer MoS_2_ films (d) grown at 900°C on α‐Al_2_O_3_, (e) grown at 1000°C on α‐Al_2_O_3_, and (f) grown on α‐Al_2_O_3_ and subsequently transferred to SiO_2._ The scale bars are 10 µm.

Sapphire surface reconstruction at elevated growth temperatures leads to non‐uniform charge doping of MoS_2_. To evaluate this heterogeneity, we first employ Raman spectroscopy, leveraging the sensitivity of the MoS_2_ out‐of‐plane $A_{1}^{\prime }$ mode to substrate‐induced charge doping [33]. Raman spectra (Figure 2a,b) collected over a 2500 µm^2^ area of MoS_2_ films grown at 900 °C and 1000 °C following 532 nm excitation exhibit characteristic in‐plane (*E*′ ≈ 385 cm–^1^) and out‐of‐plane ($A_{1}^{\prime }$ ≈ 405 cm^−1^) vibrational modes of monolayer MoS_2_ [39]. Compared to films grown at 900 °C, MoS_2_ films grown at 1000 °C display a markedly heterogeneous Raman response, particularly in the $A_{1}^{\prime }$ peak frequency distribution (Figure 2d,e). For example, some regions of the 1000 °C MoS_2_ film exhibit broad and asymmetric $A_{1}^{\prime }$ peaks (Figure 2b and Figure S4), similar to those previously reported for MoS_2_ flakes transferred onto strongly interacting substrates like Au [40]. Because the $A_{1}^{\prime }$ mode of MoS_2_ is sensitive to charge doping due to electron‐phonon coupling [41], these observations are consistent with spatial variations in carrier density across the 1000°C‐grown film. To quantitatively separate the contributions of strain and charge doping, we adopt the established relationships between strain (ε), electron concentration (*n*), and MoS_2_ *E*′ and $A_{1}^{\prime }$ phonon frequencies, given by [33]:

(1) $$\Delta \omega_{E}=-2\gamma_{E}\omega_{0}^{E}\varepsilon +k_{n}^{E}n$$

(2) $$\Delta \omega_{A}=-2\gamma_{A}\omega_{0}^{A}\varepsilon +k_{n}^{A}n$$

where $\omega_{0}^{E}$ and $\omega_{0}^{A}$ are the frequencies of the *E*′ and $A_{1}^{\prime }$ modes in the absence of strain and doping, γ_*E*_ and γ_*A*_ are Grüneisen parameters equal to 0.86 and 0.15 for the *E*′ and $A_{1}^{\prime }$ modes, and $k_{n}^{E}$ and $k_{n}^{A}$ describe how charge doping shifts the $E^{`}$ and $A_{1}^{\prime }$ modes according to

(3) $$k_{n}^{E}=-0.33\frac{cm^{-1}}{10^{13}/cm^{2}}\;\mathrm{and}\;k_{n}^{A}=-2.22\frac{cm^{-1}}{10^{13}/cm^{2}}$$

From Equations (1) and (2), we estimated the changes in strain and charge doping within our MoS_2_ films from the Raman peak positions (see Section S3). For the as‐grown 900°C sample, we observe a $A_{1}^{\prime }$ peak center distribution ranging from 404.7 cm^−1^ to 405.2 cm^−1^ (Figure 2d), corresponding to a change in electron concentration of ∆n ≈ 2.0 × 10^12^ cm^−2^ (Figure S5). Conversely, the as‐grown 1000°C sample (Figure 2e) exhibits $A_{1}^{\prime }$ peak center values spanning from 400.2 cm^−^ to 404.7 cm^−1^, indicative of a ∆n ≈ 2.1 × 10^13^ cm^−2^ variation in electron concentration across the mapped region (Figure S5). To determine if this variation in electron concentration originates from heterogeneous substrate‐film interactions, we mapped the Raman response of MoS_2_ films grown at 900°C and 1000°C after transfer to SiO_2_/Si (Figure 2c,f). The maps of the transferred films show a uniform $A_{1}^{\prime }\;$ peak position across the analysis area, with values varying from 402.7 cm^−1^ to 403.7 cm^−1^. This result indicates that local variations in electron concentration in the as‐grown 1000 °C sample arise from MoS_2_‐sapphire interactions. We note that the average $A_{1}^{\prime }\;$ peak position are lower in the transferred sample compared to the as‐grown sample at 900°C due to the well documented n‐type doping from SiO_2_ surfaces compared to Al_2_O_3_ [42].

Going beyond the $A_{1}^{\prime }\;$ peak, the *E*′ phonon, sensitive to lateral strain [33], also shows a more varied response in the 1000 °C versus 900°C sample (Figure S4). This variation further suggests non‐uniform substrate‐film interaction leading to heterogeneous strain across the as‐grown 1000 °C film. Furthermore, the broad vibrational features centered at ≈ 450 cm^−1^ (Figure S6) exhibit significant spot‐to‐spot variation in the as‐grown 1000°C sample, while their response remains relatively consistent for the other two films. This complex band in the 420 cm^−1^ to 470 cm^−1^ range was attributed to double resonant second‐order Raman processes [2vHS, 2LA(M), and 2LA(K)] [43]. Although less studied than the first‐order *E*′ and $A_{1}^{\prime }$ modes, recent reports suggest that these 2LA(vHs) peaks are sensitive probes of substrate‐induced strain [44]. The precise nature of the 2LA(vHs) band and its relation to MoS_2_ – substrate coupling will be the subject of further investigation.

**FIGURE 3:**
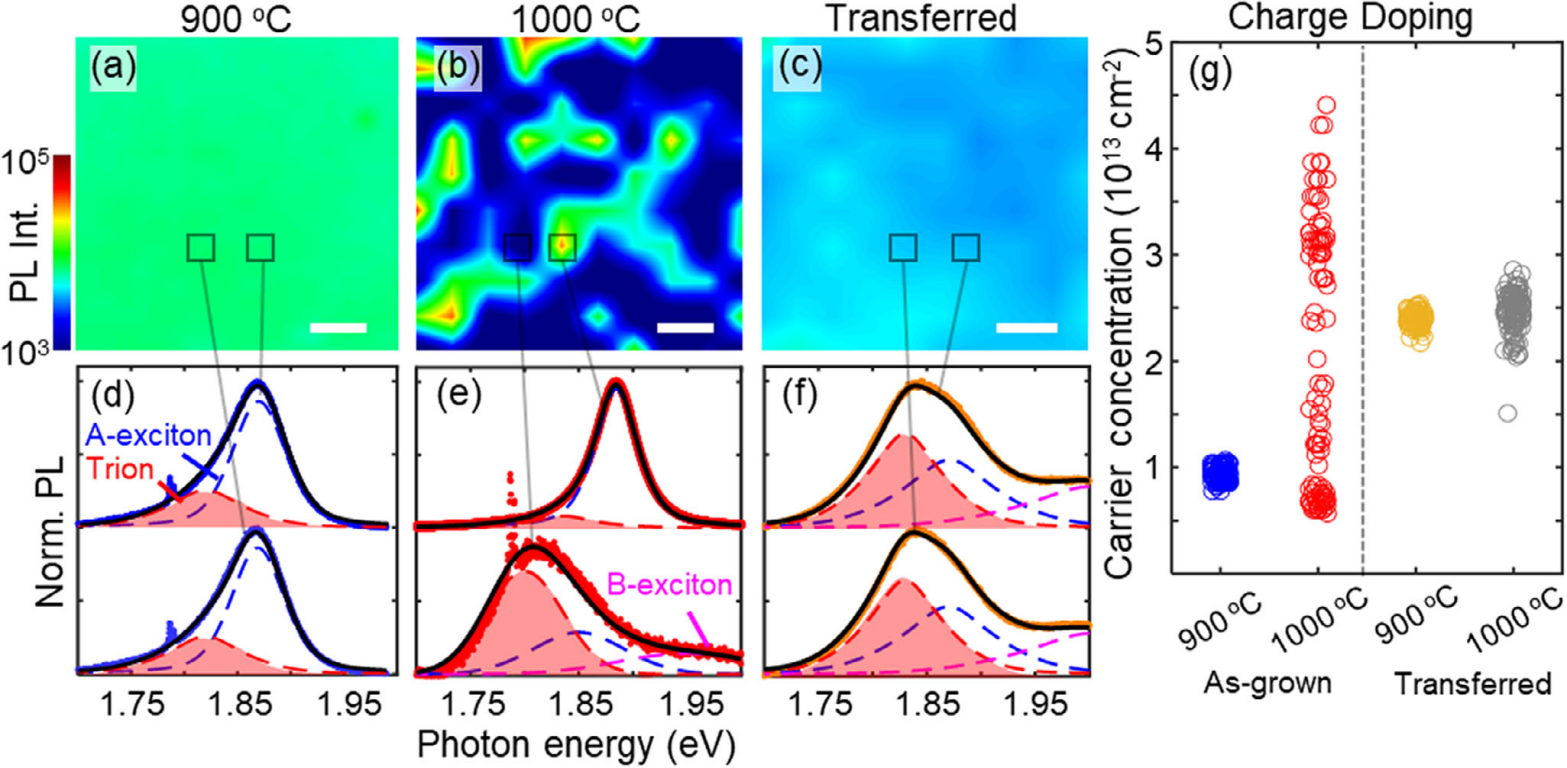
Impact of sapphire reconstruction on MoS_2_ photonic properties. Photoluminescence (PL) intensity maps of MoS_2_ films (a) grown at 900°C on α‐Al_2_O_3_, (b) grown at 1000°C on α‐Al_2_O_3_, and (c) grown at 1000°C on α‐Al_2_O_3_ and subsequently transferred to SiO_2_/Si. The scale bars are 10 µm. (d–f) Normalized PL spectra collected from the regions indicated on the PL maps. The spectra are fit with two pseudo‐Voigt curves to determine the contribution of trions (red dashed line) and A‐excitons (blue dashed line) to the PL spectra. In some regions of the 1000°C samples, a third pseudo‐Voigt curve (magenta dashed line) was used to determine the contribution of B‐excitons to the PL spectra. The sharp, low‐intensity peaks at ≈ 1.78 eV present in the as‐grown samples originate from Cr^3+^ impurity ions in the sapphire substrate. (g) PL‐derived carrier concentrations obtained from the PL spectra using a mass action model described in the supporting information.

Substrate‐induced charge doping affects the photonic properties of MoS_2_ by enhancing nonradiative recombination through trion formation, which quenches MoS_2_ emission [35]. This effect is evident when comparing the PL intensity maps (Figure 3a–c) of as‐grown and transferred MoS_2_ films collected over a 2500 µm^2^ region following optical excitation at 532 nm. PL spectra collected at select spots across the maps appear in Figure 3d–f. Notably, PL from the 1000°C as‐grown MoS_2_ film (Figure 3a and Figure S7) varies markedly across the examined area, in contrast to films grown at 900°C (Figure 3b and Figure S7). We note that although growth at 1000°C produces larger MoS_2_ grains (Figure 1), which would be expected to locally enhance PL by reducing grain‐boundary‐mediated nonradiative recombination, the PL measurements in Figure 3a and Figure S7 suggest that this effect is largely offset by increased sapphire surface reconstruction and the associated charge doping at higher growth temperatures. Time‐resolved PL measurements (Figure S8) further support substrate‐induced charge doping as the mechanism responsible for heterogeneous PL in the as‐grown 1000°C MoS_2_ film on sapphire. We fit the data shown in Figure S8 with exponential functions to obtain exciton lifetimes for films grown at 900°C and 1000°C (fit parameters in Table S1). From these fits, we obtain average PL lifetimes of 65 ps and 30 ps for the 900°C and 1000°C films, respectively. We attribute the reduced lifetime in the 1000°C film to enhanced nonradiative recombination, consistent with increased substrate‐induced charge doping and the associated trion formation reported in prior studies [34, 36, 45, 46].

Transferring the film to a pristine SiO_2_/Si substrate reduces variations in PL intensity (Figure 3c; Figure S7), further suggesting that the heterogeneous PL observed in the as‐grown 1000 °C MoS_2_ film arises from non‐uniform charge doping from the underlying c‐sapphire substrate. To quantitatively assess the variations in carrier concentration across the MoS_2_ films, we fit the PL spectra collected across the mapped regions (Figure S7) with two pseudo‐Voigt curves separated by ≈ 35 meV to determine the contribution of trions (red‐dashed line, Figure 3d–f) and neutral A‐excitons (blue dashed line, Figure 3d–f) to the overall emission. We note that emission from B‐excitons at ≈ 2 eV is also observed in some regions of the as‐grown and transferred 1000°C films, possibly due to more sulfur‐site defects [47] or increased charge doping [48] compared to films grown at 900°C. Using a mass action model (see Section S4), we find from the intensity ratio of the trion and A‐exciton peaks that the electron density within the as‐grown 1000 °C film varies by as much as ∆n ≈ 4 × 10^13^ cm^−2^ (Figure 3g). However, this variation reduces by almost a factor of seven to ∆n ≈ 6 × 10^12^ cm^−2^ after transferring the MoS_2_ film to SiO_2_/Si. This trend is consistent with our Raman measurements (Figure 2) and further demonstrates that substrate‐induced charge doping, rather than variations in MoS_2_ sample quality, is responsible for heterogeneous emission in the as‐grown 1000 °C MoS_2_ film on sapphire. We note that physically bound and electronically inactive trapped charges at the MoS_2_/substrate interface may influence the electron concentrations extracted from Raman and PL measurements. Therefore, electron densities obtained from transport measurements, as discussed in detail below, do not capture the presence of these charges.

**FIGURE 4:**
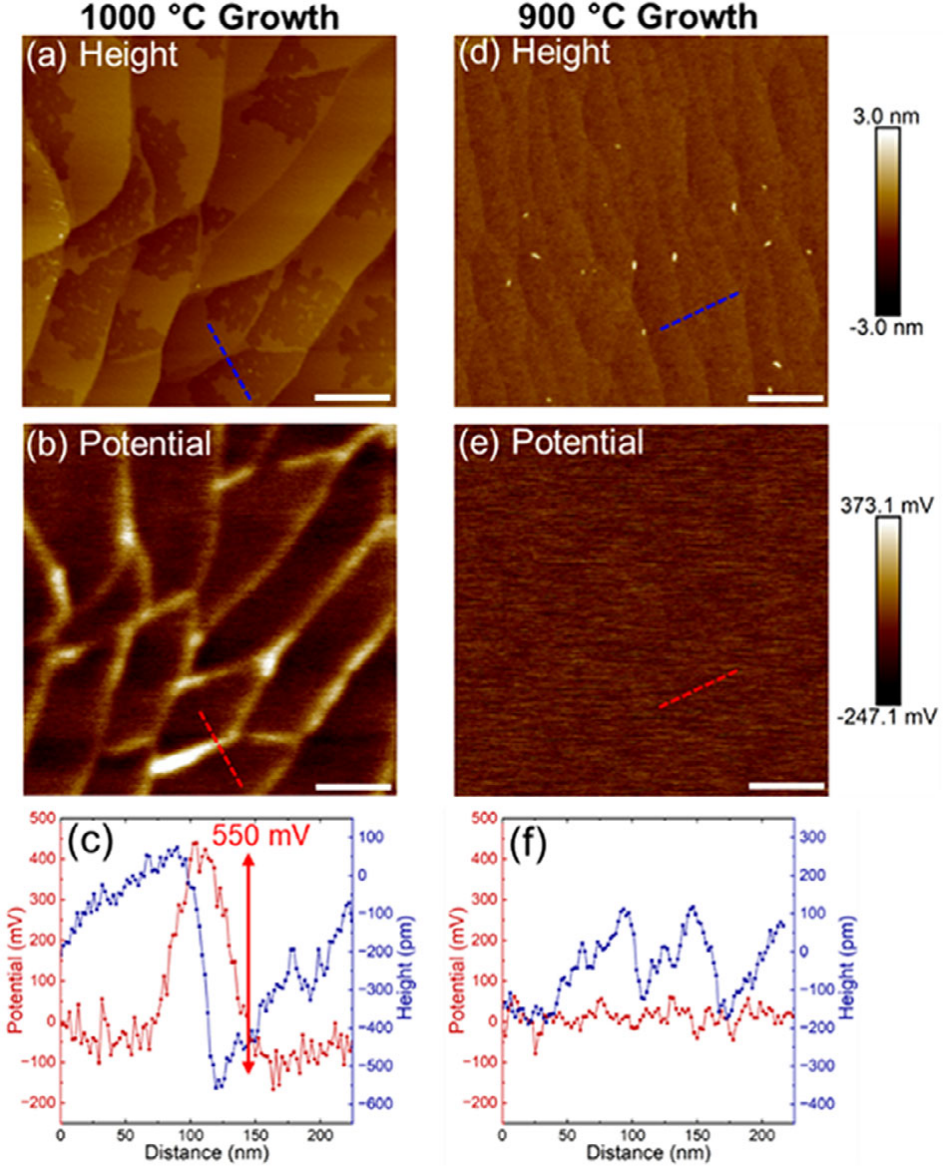
Effect of step‐edge formation on MoS_2_ electronic properties. AFM topography (a) and KPFM surface potential (b) of a monolayer MoS_2_ sample grown on α‐Al_2_O_3_ at 1000°C showing modulation of Fermi level at the step edges of the underlying substrate. AFM topography (d) and KPFM surface potential (e) of a monolayer MoS_2_ sample grown on α‐Al_2_O_3_ at 900°C showing that step edges have little effect on the film's Fermi level. The scale bars are 200 nm. Line profiles of height channel (blue) and potential channel (red) for MoS_2_ samples grown at (c) 1000°C and (f) 900°C.

Kelvin‐probe force microscopy offers direct evidence that the modification in substrate topography during growth impacts charge doping within the MoS_2_ epilayer. Specifically, KPFM offers spatially resolved measurements of surface‐potential variations, which directly correspond to local shifts in the MoS_2_ Fermi level (see Section S6) [49]. KPFM scans (Figure 4) of a MoS_2_ film grown at 1000°C reveal that the step edges of the underlying α‐Al_2_O_3_ substrate cause local variations in the film's surface potential (Figure 4a,b), with the step edges exhibiting a higher surface potential (corresponding to a higher Fermi level) compared to the terrace region. We highlight this variation in surface potential by drawing a line profile over a step edge (Figure 4c), which shows that the local variation in surface potential can reach values of up to approximately 550 mV. Similar variations in surface potential have been measured from KPFM scans of WSe_2_ films grown on α‐Al_2_O_3_ substrates and attributed them to the coupling between the sapphire substrate and the WSe_2_ film [4]. Interestingly, KPFM analysis of a MoS_2_ film grown at 900°C (Figure 4d,e) reveals reduced surface potential variations compared to the 1000 °C analog. A line profile over two step edges in the 900 °C sample (Figure 4f) demonstrates that the surface potential does not vary in relation to the step edges. The difference in the spatial uniformity of the surface potential between films grown at 900 °C and 1000 °C implies that substrate‐film coupling occurs predominantly at high growth temperatures, where the step‐and‐terrace morphology of the sapphire substrate is dramatically modified. Although the present study is limited to c‐plane sapphire, we acknowledge that high‐temperature growth on other sapphire cuts (m‐plane, a‐plane, and r‐plane) and resultant effects on the properties of 2D epilayers is an important topic. For example, Bansal et al. reported that hBN films could be successfully transferred from a‐plane sapphire but not from c‐plane sapphire, which they attributed to a higher degree of chemical bonding and consequently stronger film‐substrate adhesion on the c‐plane surface [21]. A thorough study of these substrates is beyond the scope of this work and will be addressed in future studies.

Examination of the sapphire surface after transfer of the MoS_2_ epilayer provides further evidence of MoS_2_‐sapphire coupling at step‐edges. In the 1000°C case, residue is leftover along the step edges of the sapphire substrate after transfer (Figure S9). X‐ray photoelectron spectroscopy examination of the substrate after transfer shows the presence of Mo and S in the residue (Figure S9), suggesting that removing the layer from the step edge areas is more challenging due to its stronger bonding with the substrate. Conversely, for the 900°C sample substrate, the surface after layer transfer is residue‐free (Figure S9), suggesting reduced coupling between the MoS_2_ layer and the underlying substrate. These observations demonstrate that growth‐induced interfacial interactions at elevated temperatures can modify film adhesion to the underlying substrate. Such adhesion is expected to impact the yield and uniformity of layer transfer processes that are critical for the integration of 2D materials into Front End of Line (FEOL) technologies [50, 51].

**FIGURE 5:**
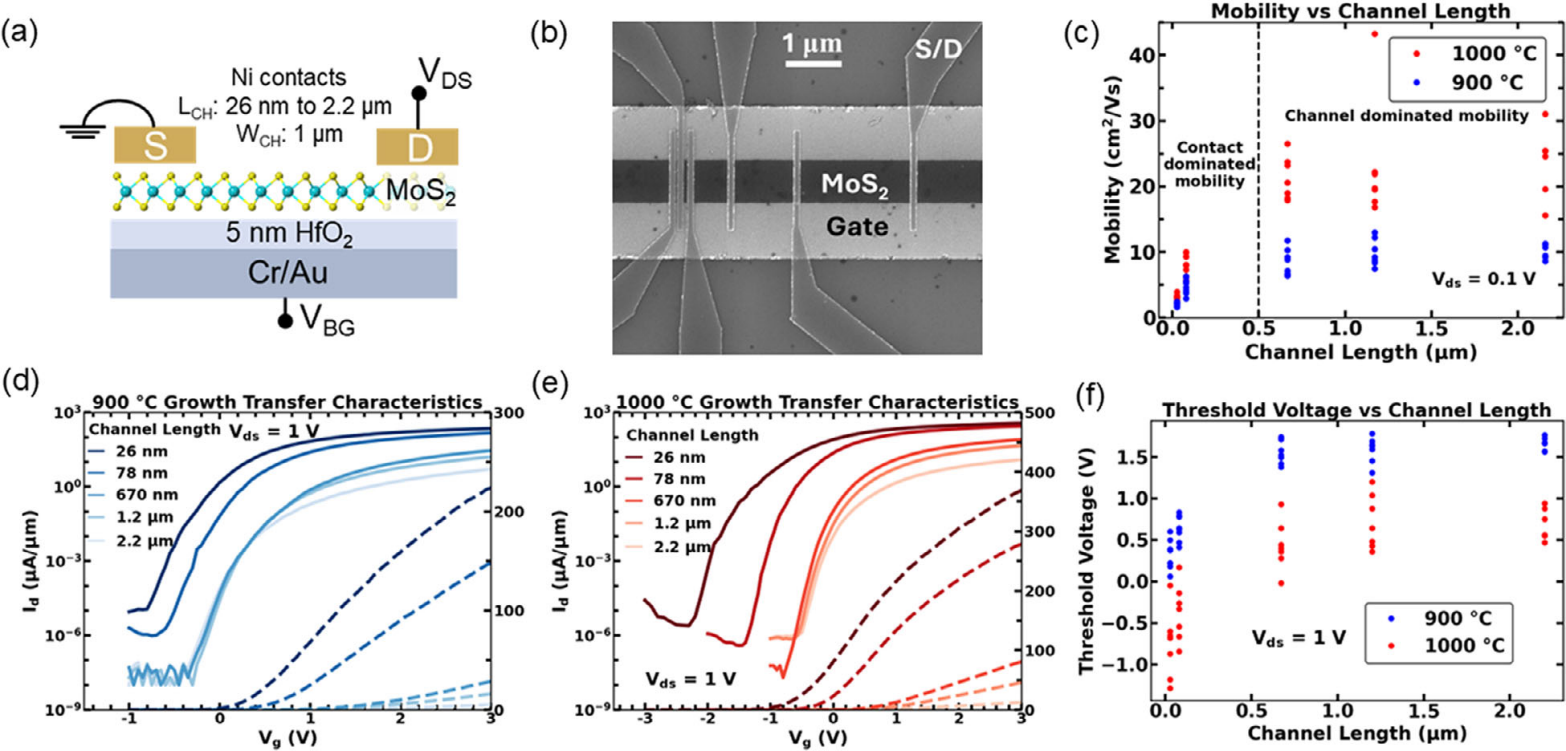
Transport characteristics of BGFETs fabricated on MOCVD‐grown monolayer MoS_2_. (a) Schematic of an ML MoS_2_ BGFET device showing Ni S/D contacts and 5 nm HfO_2_/5 nm Cr/15 nm Au gate. (b) Scanning Electron Microscopy (SEM) image of a representative BGFET device in a TLM structure with channel lengths ranging from 2.2 µm to 26 nm. (c) Threshold voltages measured vs. channel length for growth temperatures of 900°C and 1000°C, extracted with *V_ds_* = 1 V. (d) Transfer characteristics at *V_ds_* = 1 V of a representative TLM of 900°C grown ML MoS_2_ with log (solid) and linear (dashed) scaling. (e) Transfer characteristics at *V_ds_* = 1 V of a representative TLM of 1000°C grown ML MoS_2_ with log (solid) and linear (dashed) scaling, note the change in x and y axis compared to d). (f) Field‐effect mobility vs. channel length for growth temperatures of 900°C and 1000°C, extracted from *g_max_* of the *I_d_‐V_gs_* characteristics at *V_ds_* = 0.1 V. Regions of channel and contact dominance on mobility have been demarcated.

Transport measurements on BGFETs devices demonstrate that films grown at 1000°C consistently outperform those at 900°C in terms of mobility and drain current, suggesting superior material quality. To explore these differences in detail, we fabricated BGFETs (Figure 5a) by transferring MoS_2_ monolayers grown at 900°C and 1000°C on α‐Al_2_O_3_ to local bottom‐gate (LBG) substrates (details in the Methods Section). After transferring, we etched the MoS_2_ into 1 µm wide strips and deposited Ni source/drain (S/D) contacts patterned in a transfer length method (TLM) structure with channel lengths (*L_ch_*) ranging from 2.2 µm to 26 nm to ascertain channel length dependencies (Figure 5b).

Devices made on 900°C grown films exhibited a clear scaling trend between the current and channel length down to 26 nm *L_ch_* (Figure 5d), indicating contact resistances do not overwhelm channel resistances for all channel lengths. Drain currents reach close to 300 µA/µm at a drain voltage of 1 V for the shortest channel lengths. A leftward shift of ≈ 1 V toward negative threshold voltage (*V_t_*) values is observed for decreasing *L_ch_*, a feature that hints at short‐channel effects for the shortest transistors. This observation is somewhat supported by our finding of (i) a degrading inverse subthreshold slope (Figure S10a) and (ii) an increasing drain induced barrier lowering (DIBL) with decreasing channel length (Figure S10b), although the variation in Figure S10a,b is too substantial to be conclusive. The investigation into the observed shifts in threshold voltage, which are not anticipated from an electrostatic perspective, remains ongoing but is beyond the scope of the current study.

Conversely, devices made on 1000°C films show larger drain currents compared to 900°C BGFETs (Figure 5e), reaching 400 µA/µm at *V_gs_* = 3 V for the shortest *L_ch_*. The linear *I_d_‐V_ds_* plots demonstrate that currents begin to saturate at large *V_gs_* when *L_ch_* is small, highlighting the impact of the contact resistance. An even more pronounced *V_t_* dependence on *L_ch_* is observed compared to the 900°C films, resulting in *V_gs_* values as small as −3 V to reach the deep off state of some devices. Moreover, 1000°C films show a leftward *V_t_* shift of about 1 V compared to 900°C films of the same *L_ch_* (Figure 1c). As discussed above, this threshold voltage roll‐off is still under investigation. For each TLM structure a linear dependence is observed between the total resistance *R_T_* = *V_ds_* /*I_d_* and *L_ch_* with *V_ds_* = 1 V. Device resistances are characterized using a linear regression where the slope equates to the sheet resistance (*R_sh_*) and the intercept equates to double the contact resistance (2*R_c_*), where channel resistance *R_ch_* = *R_sh_* *L_ch_*. Figure S10c,d shows *R_sh_* and *R_c_* versus carrier density *n*  =  *C_ox_V_ov_*, where *V_ov_* =  *V_g_* − *V_t_*. Smaller R_sh_ values are observed for 1000°C films, while *R_c_* appears independent of film growth temperature. At a carrier density of 10^13^ cm^−2^ (*V_ov_* ≈ 1 V), 900°C films exhibit mean *R_c_* of 3.3 kΩ µm ± 1.9 kΩ µm, and mean *R_sh_* of 70 kΩ/□ ± 18 kΩ/□, so that *R_ch_* < 2*R_c_* for *L_ch_* < 94 nm. At the same density, 1000°C films exhibit mean *R_c_* of 2.9 kΩ µm ± 0.85 kΩ µm, and mean *R_sh_* of 29 kΩ µm /□ ± 6 kΩ µm /□, so that *R_ch_* < 2*R_c_* for *L_ch_* < 200 nm. Field effect mobilities were extracted from the maximum transconductance (g_max_) of the *I_d_‐V_gs_* characteristics at *V_ds_* = 0.1 V. We note that for large *L_ch_*, mobilities are relatively constant with *L_ch_*. Notably, 1000°C films exhibit mobility values (≈ 16 cm^2^V^−1^s^−1^ to 43 cm^2^ V^−1^s^−1^ at *V_ds_* = 0.1 V) consistent with MOCVD‐grown TMD FETs [18] and ≈ 2x higher than their 900°C analogs. This result is consistent with the ≈ 2x larger grain boundary density in 900°C films, suggesting that grain boundary scattering is the primary scattering mechanism in these films. For channel lengths smaller than the grain boundary size, higher mobility is expected. However, this effect is outweighed by contact resistance, which dominates in the shortest channel devices. 900°C films, which exhibit V_t_ values that are more positive for the shortest channel lengths than 1000°C films, exhibit smaller currents than 1000°C films at the same *V_g_*. This higher current may result from the larger achievable overdrive voltage in the latter. However, even when accounting for these threshold voltage differences, 1000°C films still outperform the 900°C analogs due to larger grain sizes and, subsequently, higher carrier mobilities. These results further suggest that heterogeneous optical properties observed across the 1000°C film (Figures 2 and 3) reflect non‐uniform substrate‐film coupling and not the quality of the as‐grown film.

## 3 Conclusion

We have demonstrated that sapphire surface reconstruction strongly influences the properties of MoS_2_ films. AFM and KPFM measurements show that step‐edge formation at elevated growth temperatures (T = 1000°C) significantly modifies the MoS_2_/sapphire interface, producing local variations in surface potential of up to ≈ 550 mV across step edges. Raman and PL measurements confirm that these step edges introduce considerable inhomogeneities in strain and charge doping within films grown at 1000°C. Notably, these pronounced changes in substrate–film interaction emerge within a relatively narrow growth‐temperature window (900°C to 1000°C), highlighting the sensitivity of TMD‐sapphire interfacial coupling to modest variations in synthesis conditions. Transferring the films to SiO_2_/Si reduces these inhomogeneities, indicating that variations in Raman peak positions and PL intensities in as‐grown films do not necessarily reflect intrinsic material quality but can arise from non‐uniform substrate‐induced charge doping. Statistical data from multiple BGFET devices fabricated across the 1000°C sample further support this conclusion by exhibiting uniform threshold voltage, on‐current, and mobility values. These results stress the importance of substrate reconstruction during growth as a previously underappreciated variable that impacts the optical and electronic properties of the epilayer. Moreover, these findings caution against an overreliance of standard optical assessment techniques such as Raman and PL spectroscopy to determine TMD sample quality while the TMD is still on its growth substrate, as substrate reconstruction during synthesis can lead to heterogeneous TMD‐substrate interactions that affect Raman peak positions and PL intensities. Beyond optical metrology, substrate–film coupling also directly influences adhesion strength and, in turn, can impact the yield and uniformity of layer transfer processes essential for wafer‐scale 2D material device integration.

## 4 Methods

### 4.1 MOCVD Growth

We used a home‐built metal‐organic chemical vapor deposition (MOCVD) reaction to grow MoS_2_ films. In this set‐up, we place a Mo(CO)_6_ (99.99% purity, Sigma–Aldrich) powder into a stainless steel bubbler with an internal pressure of 98 kPa. We then heat the bubbler to 24°C. We control the concentration of Mo precursor in the MOCVD reactor by regulating the flow of a high‐purity H_2_ carrier gas through the bubbler using a mass flow controller. Sulfur was introduced into the reactor by injecting H_2_S (99.5%, Sigma–Aldrich) through a mass flow controller.

Before growth, c‐plane sapphire substrates are loaded into the reactor hot zone and annealed at 300°C for 15 min at a base pressure of 10^−^^4^ kPa to desorb moisture. Next, we filled the MOCVD chamber with Ar gas to a growth pressure of 6.7 kPa before heating the chamber at a rate of 50°C/min to 900°C or 1000°C. Subsequently, we delivered precursors to the sapphire substrate surface by flowing Ar into the chamber. A three‐step nucleation, ripening, and lateral growth process was used to grow uniform MoS_2_ films. First, 3.0 × 10^−4^ mol/min (7.5 sccm) H_2_ carrier gas was flown through the Mo(CO)_6_ bubbler and 1.6 × 10^−3^ mol/min (40 sccm) H_2_S are flown for 2 min to create nuclei on the substrate surface. The nuclei are ripened into MoS_2_ domains by stopping Mo(CO)_6_ delivery to the substrate while maintaining a steady H_2_S flow rate for 10 min. Following this ripening stage, MoS_2_ domains are laterally grown by flowing 1.5 × 10^−4^ mol/min H_2_ carrier gas through the Mo(CO)_6_ bubbler and 1.6 × 10^−3^ mol/min H_2_S into the reactor for 24 min.

### 4.2 Layer Transfer

The layer transfer process starts with coating the MoS_2_/sapphire samples with PMMA (Poly(methyl methacrylate), Micro Chem. 950K A6) using a two‐step spin coating technique. Initially, the samples are spun at 52 rad/s for 15 s, followed by 471 rad/s for 45 s. The coated stack is then left in ambient conditions overnight to ensure complete evaporation of the solvent from the PMMA layer. Following this, a layer of thermal release tape (TRT, Semiconductor Corp., with a release temperature of 170°C) was gently applied to the PMMA/MoS_2_/sapphire stack. The stack was then immersed in a sonication bath containing deionized (DI) water at 80°C for 15 min. This sonication step aids in detaching the growth substrate from the stack. After substrate removal, the remaining MoS_2_/PMMA/TRT stack is dried using nitrogen gas to remove any residual water on the MoS_2_ surface. For transferring the MoS_2_ film to the target substrate, the dried stack was carefully stamped onto the target substrate. Ensuring proper adhesion of the film to the new substrate is crucial. The TRT was then removed by heating it above its release temperature (180°C) on a hot plate. Finally, the PMMA layer was dissolved by soaking the sample in acetone for 8 h. The sample is then rinsed with isopropyl alcohol (IPA) and dried on a hot plate set at 80°C to complete the transfer process.

### 4.3 Raman and PL Spectroscopy

We used a Horiba Jobin‐Yvon LabRam Evolution Raman microscope (Horiba, Edison, NJ) for both PL and Raman measurements. We focused the output of a continuous‐wave 532 nm laser onto the sample using a 100× 0.95 NA objective (Olympus). We collected the Raman and PL signals with the same objective before detecting them using a charge‐coupled device (CCD) camera (Back‐Illuminated Synapse, Horiba, Edison, NJ) placed after a spectrometer. For Raman maps, we acquired Raman spectra using an 1800 lines per millimeter grating in 5 µm steps with a 5 sec acquisition time following 0.41 kW/cm^2^ optical excitation. We obtained PL maps using the same power density and step size as Raman measurements. All Raman and PL measurements were obtained in air at room temperature after Si spectral calibration.

Time‐resolved photoluminescence (TRPL) measurements were performed using a custom‐built PL microscope described previously [34]. A 1030 nm, 1 MHz Yb:KGW laser (Carbide, Light Conversion Ltd., Vilnius, Lithuania) pumped an optical parametric amplifier (OPA). The 900 nm OPA output was frequency‐doubled in a β‐barium borate (BBO) crystal to generate 450 nm excitation pulses, delivering 5 pJ per pulse at the sample. A 40×, 0.75 NA objective both focused the excitation beam onto the sample and collected the emitted PL. The collected PL was dispersed by a spectrograph and detected with a streak camera (Hamamatsu C14831‐130).

### 4.4 Atomic Force and Kelvin Probe Force Microscopy (AFM and KPFM)

Atomic force microscopy is performed using Bruker Dimension Icon instrument equipped with a ScanAsyst‐Air (*k* = 0.4 N/m) tip in tapping mode. During high‐resolution scans, the tip scan rate is set to 0.5 Hz while maintaining a peak force set point of 1.00 nN and a peak force frequency of 2 kHz. KPFM data was acquired using PFQNE‐AL probes in peak force KPFM mode. For surface potential measurements, the light height was set at 35 nm, and a 10 V AC bias was applied to the tip.

### 4.5 BGFET Fabrication

Bottom‐gate field‐effect transistors (BGFETs) were fabricated from monolayer (ML) MoS_2_ films grown at 900°C and 1000°C. First, local bottom‐gate (LBG) substrates were fabricated. Patterned 5 nm/15 nm Cr/Au gates were formed on a SiO_2_/p++ Si substrate using e‐beam lithography and e‐beam evaporation. Next, a 5.5 nm HfO_2_ gate dielectric was conformally grown using atomic layer deposition (ALD) at 200°C to finish the LBG substrate. The MoS_2_ films were then transferred from their sapphire growth substrates to the LBG substrate. The film was patterned into 1 µm wide strips above the LBGs using again e‐beam lithography and a Cl_2_/O_2_ plasma reactive ion etch (RIE). Finally, nickel source/drain (S/D) contacts of 25 nm in height were defined by e‐beam lithography and deposited using e‐beam evaporation. These contacts were patterned in TLM structures with channel lengths ranging from 2.2 µm to 26 nm.

## Conflicts of Interest

The authors declare no conflicts of interest.

## Supporting information




**Supporting File**: smll73090‐sup‐0001‐SuppMat.docx.

## Data Availability

The data that support the findings of this study are available from the corresponding author upon reasonable request.
