# Supplementary material for: Impact of Sapphire Substrate Reconstruction on the Structural, Electronic, and Photonic Properties of MoS2
Source: Small. 2026 Mar 11;22(26):e11179. doi: 10.1002/smll.202511179 (PMC13155076; doi:10.1002/smll.202511179)
Supplement: Supplementary file 1 — Supporting File: smll73090‐sup‐0001‐SuppMat.docx. [file SMLL-22-e11179-s001.docx]

Supporting Information

**Impact of Sapphire Substrate Reconstruction on the Structural, Electronic, and Photonic Properties of MoS_2_**

Riccardo Torsi,^1†^ Kyle T. Munson,^1†^ Daniel Eppler,^2^ Jo Laura,^3^ Furkan Turker,^1^ Maxwell Feidler,^1^ Joerg Appenzeller,^2^ Yu-Chuan Lin,^4^ Joshua A. Robinson,^1,4,5,6#^

1. Department of Materials Science and Engineering, The Pennsylvania State University, University Park, Pennsylvania 16802, United States
2. Department of Electrical and Computer Engineering, Purdue University, West Lafayette, IN 47907, United States
3. Department of Chemistry, The Pennsylvania State University, University Park, Pennsylvania 16802, United States
4. Department of Materials Science and Engineering, National Yang Ming Chiao Tung University, Hsinchu City 300, Taiwan
5. Materials Research Institute, The Pennsylvania State University, University Park, PA, 16802, United States
6. Department of Physics, The Pennsylvania State University, University Park, Pennsylvania 16802, United States

# Corresponding Author: [jar403@psu.edu](mailto:jar403@psu.edu)

† Denotes equal contribution

### Author Note:

### Riccardo Torsi is now at the Physical Measurement Laboratory, National Institute of Standards and Technology, Gaithersburg, Maryland 20899, United States

Kyle T. Munson is now an NRC Postdoc at the Electronics Science and Technology Division, United States Naval Research Laboratory, Washington, DC 20375, United States

Section I. AFM topography of early stages of film growth and additional statistical analysis on grain morphology

Section II. AFM topography of SiO_2_/Si substrates

Section III. Raman characterization of MoS_2_ films

Section IV. PL Characterization of MoS_2_ films

Section V. Characterization of sapphire substrates after MoS_2_ sample transfer

Section VI: Kelvin Probe Force Microscopy Measurements:

Section VII: Channel Length effects of BGFETs fabricated on MOCVD-grown monolayer MoS_2_

Section VIII. References

**Section I. AFM topography of early stages of film growth and additional statistical analysis on grain morphology**


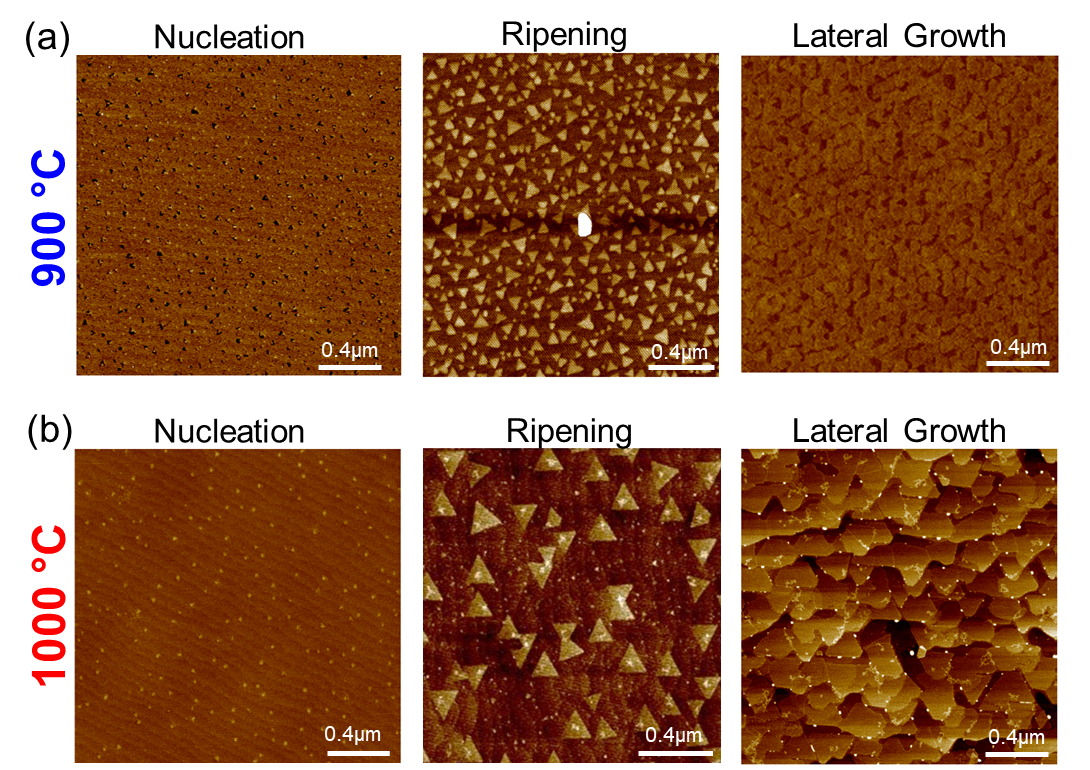


**Figure S1.** Atomic Force Microscopy images showing the morphological evolution of the MoS_2_ films at the different stages of growth (nucleation, ripening, and lateral growth) for films grown at (a) 900 ^o^C and (b) 1000 ^o^C.

We provide additional atomic force microscopy (AFM) scans comparing the morphological evolution of a monolayer MoS_2_ films synthesized at 900 °C and 1000 °C in **Figure S1a** and **Figure S1b**, respectively. Following the initial nucleation step, the nucleation density decreases from ≈ 118 nuclei/µm^2^ at 900 °C to ≈ 55 nuclei/µm^2^at 1000 °C. This reduction is consistent with a lower sticking factor at higher temperature and has been demonstrated in other reports of CVD grown TMDs.^[1]^ After the ripening step, both cases show that some of the nuclei develop into triangular domains. However, the initial difference in nucleation density is reflected in a substantially lower domain density for the 1000 **°**C growth compared to the 900 **°**C case. Notably, even at this early stage, the domains grown at 1000 **°**C are larger and show improved alignment uniformity compared to the lower temperature sample. This enhanced domain size and improved alignment is maintained throughout the monolayer growth until the domains start to merge and the film coalescences. Importantly, in the context of sapphire substrate modification, the morphological evolution of the 1000 **°**C film suggests that the substrate reconstruction occurs continuously during the growth process and not during the ramp up.


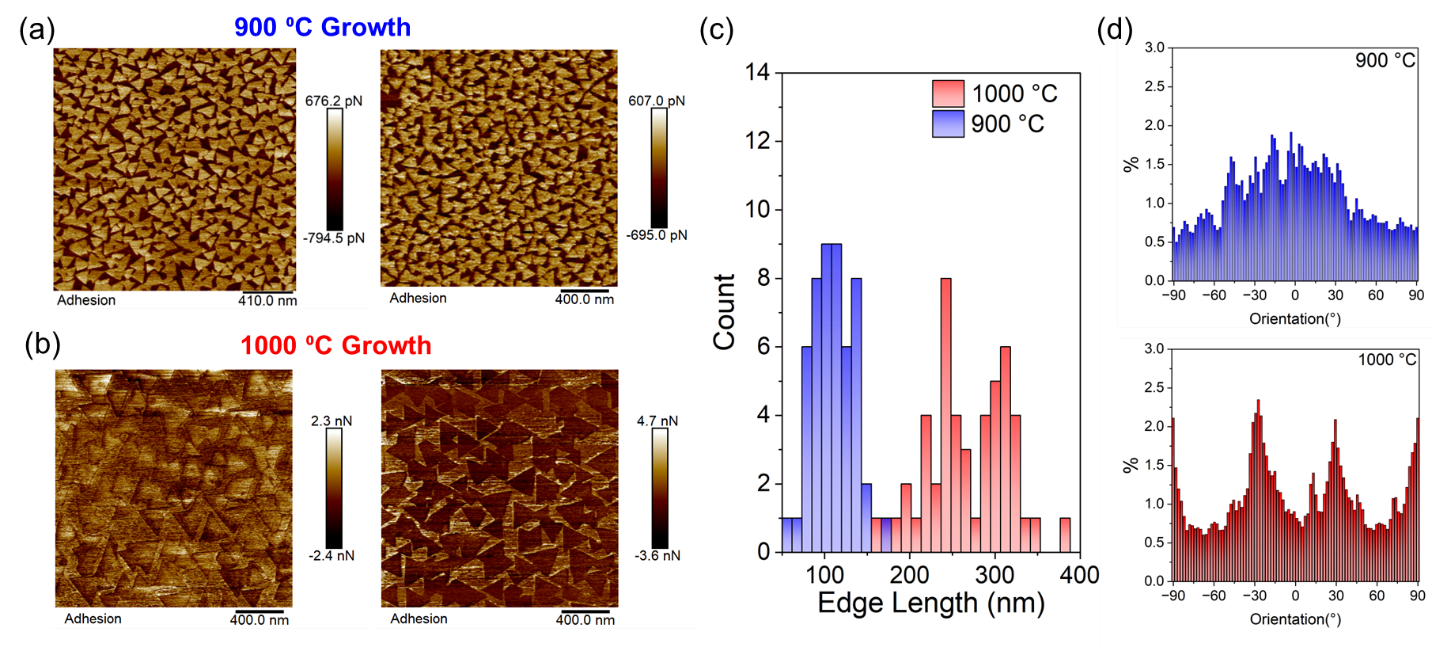


**Figure S2.** Statistical Analysis of grain morphology for MoS2 films grown at 900 °C and 1000 °C. Atomic force microscopy scans of MoS_2_ films grown at (a) 900 °C and (b) 1000 °C acquired prior to coalescence. The adhesion channel is shown to enhance contrast between MoS_2_ domains and substrate. (c) Distribution of edge length measurements and (d) domain orientation comparing films grown at 900 °C and 1000 °C.

We provide statistical analysis of grain morphology differences between films grown at 900 °C (**Figure S2a**) and 1000 °C (**Figure S2b**). AFM scans are acquired prior to monolayer coalescence to reveal the underlying grain structure. For a more reliable comparison between the two growth conditions, the adhesion channel is shown rather than the height channel, as it provides enhanced contrast of grain boundaries and domain morphology. **Figure S2c** shows distribution of edge length measurements extracted from the corresponding AFM images. Higher growth temperatures lead to an increase in average grain edge length from 109 nm ± 23 nm to 267 nm ± 48 nm. In addition to larger domains, higher growth temperatures also lead to an improvement in domain alignment, as shown in **Figure S2d**. The 1000 °C sample shows distinct peaks separated by 30⁰, confirming good epitaxial alignment, whereas the 900 °C sample displays a broad distribution without a particular directional preference.

**
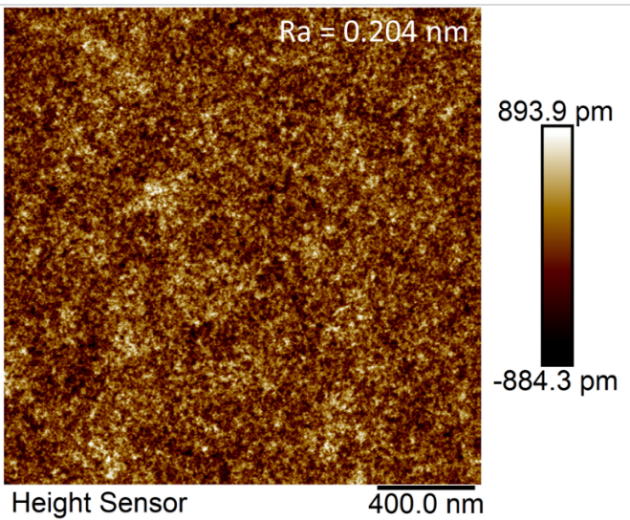
Section II.** **AFM topography of SiO_2_/Si substrates**

**Figure S3.** Atomic force microscopy scan of a SiO_2_/Si substrate. The scan reveals that SiO_2_/Si substrates lack step and terrace morphology compared to sapphire (Figure 1, main text).

**Section III.** **Raman characterization of MoS_2_ films**

**Figure S4.** Peak center and full width at half maximum (FWHM) values for the (a) in plane ( $E^{`}$) and (b) out of plane ($A_{1}^{`}$) modes of as grown and transferred MoS_2_ films collected over a 2500 µm^2^ area.


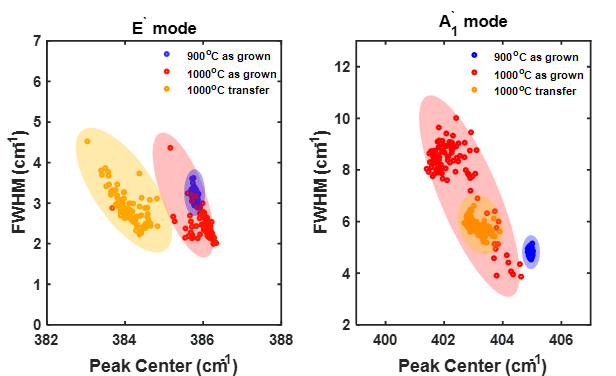


We used a model developed by Papagelis^[2]^ and Dravid^[3]^ to examine the effects of sapphire reconstruction on strain and charge doping within MoS_2_ films. As described in the main text, strain-induced shifts (∆ω) in the $E^{`}$and $A_{1}^{`}$ modes of MoS_2_ are given by $\Delta\omega=\omega_{0}- \omega=2\gamma\omega_{0}\varepsilon$, where $\omega_{0}$is a vibrational mode’s frequency in the absence of strain, $\omega$ is the mode’s frequency in a strained lattice, γ is a Grüneisen parameter, and ε is biaxial strain. Similarly, charge doping shifts the center frequency of $E^{`}$and $A_{1}^{`}$ modes by

$k_{n}^{E}=-0.33\frac{{cm}^{-1}}{{10}^{13}/{cm}^{2}}$ and $k_{n}^{A}=-2.22\frac{{cm}^{-1}}{{10}^{13}/{cm}^{2}}$.

Therefore, the relationship between strain, charge doping, and $E^{`}$and $A_{1}^{`}$ peak positions is given by,

${\Delta\omega}_{E}=-2\gamma_{E}\omega_{0}^{E}\varepsilon+k_{n}^{E}n$ (Eqn. S1)

${\Delta\omega}_{A}=-2\gamma_{A}\omega_{0}^{A}\varepsilon+k_{n}^{A}n$ (Eqn. S2)

where $\omega_{0}^{E}$ and $\omega_{0}^{A}$ are the frequencies of the MoS_2_ $E^{`}$ and $A_{1}^{`}$ modes at zero strain and doping and $n$ is electron concentration in units of 10^13^ cm^-2^. $\gamma_{E}$ and $\gamma_{A}$are Grüneisen parameters for the $E^{`}$and $A_{1}^{`}$ modes equal to 0.86 and 0.15, respectively.^[3]^ Equations S1 and S2 can be expressed in matrix form by,

$\left( \begin{matrix} {\Delta\omega}_{E} \\ {\Delta\omega}_{A} \end{matrix} \right)=\left( \begin{matrix} -2\gamma_{E}\omega_{0}^{E} & k_{n}^{E}n \\ -2\gamma_{A}\omega_{0}^{A} & k_{n}^{A}n \end{matrix} \right)\left( \begin{matrix} \varepsilon\\ n \end{matrix} \right)$ (Eqn. S3)

From Eqn. S3, we examined the effects of sapphire reconstruction on strain and charge doping density using a (𝜀-𝑛) map that describes the relationship between these parameters and MoS_2_ Raman peak positions (**Figure S5**). Because the unstrained and undoped $E^{`}$ and $A_{1}^{`}$ phonon frequencies are difficult to obtain experimentally,^[2]^ we focus our analysis on the relative changes in carrier concentration and strain across each film, rather than reporting their absolute values.

**
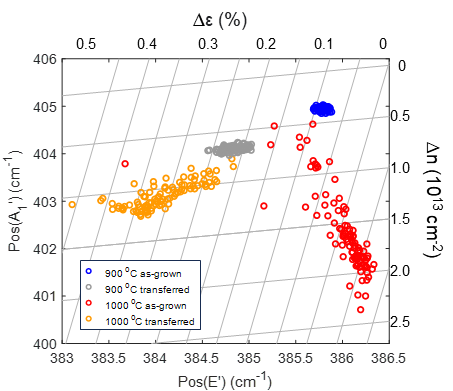
**

**Figure S5.** Raman-derived strain-charge doping (ε-n) map constructed from the linear relationship between strain, charge doping, and MoS_2_ Raman peak positions.


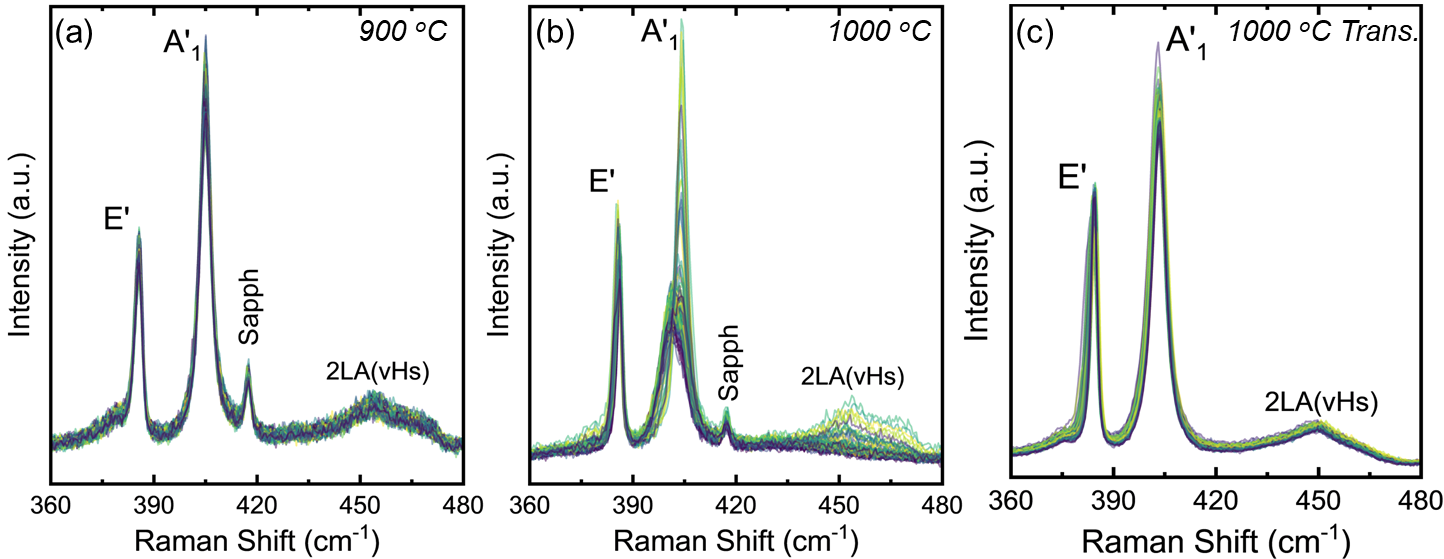


**Figure S6.** Raman spectra collected from a 2500 µm^2^ mapped area for MoS_2_ films grown on α-Al_2_O_3_ at (a) 900 °C and (b) 1000 °C. (c) Raman spectra of the 1000 °C film after transfer to SiO_2_/Si. For the 1000 °C as-grown MoS_2_ film, the double resonant second-order Raman modes in the 420 cm^-1^ to 470 cm^-1^ [2vHS, 2LA(M), and 2LA(K)] range exhibit significant spot-to-spot variation.

**Section** **IV. PL Characterization of MoS_2_ films**


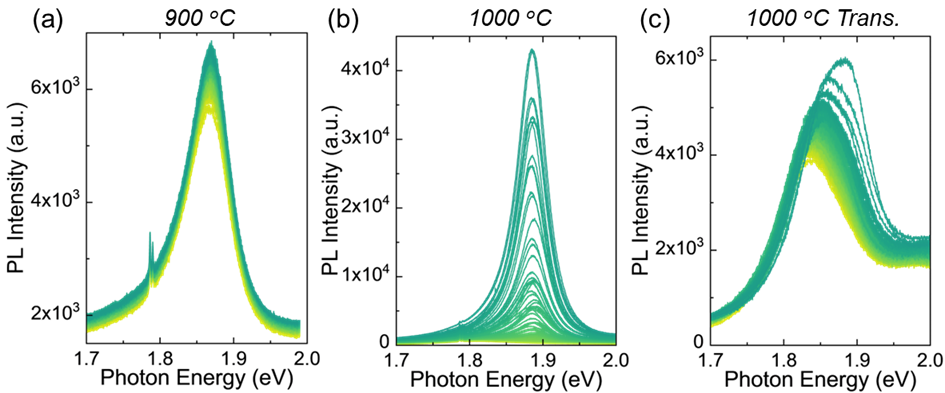


**Figure S7.** Photoluminescence (PL) spectra collected from a 2500 µm^2^ mapped area for MoS_2_ films grown on α-Al_2_O_3_ at (a) 900 °C and (b) 1000 °C. (c) PL spectra of the 1000 °C film after transfer to SiO_2_/Si. PL from the 1000 °C as-grown MoS_2_ film varies markedly across the examined area, in contrast to films grown at 900 °C or transferred to SiO_2_/Si. The sharp, low-intensity peaks at ≈ 1.78 eV present in the as-grown samples originate from Cr^3+^ impurity ions in the sapphire substrate.^[4]^

We estimated the electron density within MoS_2_ films grown on α-Al_2_O_3_ using a mass action model^[4]^ that describes the relationship between neutral excitons, trions, and excess electrons via

$\frac{N_{ex}n_{el}}{N_{tr}}= \frac{4m_{ex}m_{el}}{\pi\boldsymbol{\hbar}^{2}m_{tr}}k_{b}T\cdot exp(-\frac{E_{b}}{k_{b}T})$ (Eqn. S4)

where $N_{ex}$ is the neutral exciton population, $N_{tr}$ is the trion population, n_el_ is the doped electron density, T is temperature, $k_{b}$ is the Boltzmann constant, $E_{b}$ is the trion binding energy (≈ 35 meV), and $m_{ex}$, $m_{tr}$, and $m_{el}$ are the effective masses of excitons (0.8 m_0_), trions (1.15 m_0_), and electrons (0.35 m_0_), respectively.^[5]^ The PL intensity of excitons ${(I}_{ex})$and trions ${(I}_{tr})$ is described by

$I_{ex}=\frac{AG\gamma_{ex}}{\Gamma_{ex}+k_{tr}}$ (Eqn. S5)

$I_{tr}=\frac{k_{tr}}{\Gamma_{tr}}\cdot\frac{AG\gamma_{tr}}{\Gamma_{ex}+k_{tr}}$ (Eqn. S6)

where A is the PL collection efficiency, G is the exciton optical generation rate, $\gamma_{ex}$and $\gamma_{tr}$ are the exciton and trion radiative decay rate constants, $\Gamma_{ex}$ is the total decay rate constant for neutral excitons ($\Gamma_{ex}$= 0.002 ps^-1^),^[6]^ $\Gamma_{tr}$ is the total decay rate constant for trions ($\Gamma_{tr}=0.02 {ps}^{-1})$,^[6]^ and $k_{tr}$ is the trion formation rate constant ($k_{tr}=0.5 {ps}^{-1})$.^[4]^ When $k_{tr} \gg\Gamma_{ex}$, the PL intensity of neutral excitons and trions is given by

$I_{ex}\approx\frac{AG\gamma_{ex}}{k_{tr}}$ (Eqn. S7)

$I_{tr}\approx\frac{AG\gamma_{tr}}{\Gamma_{tr}}$ (Eqn. S8)

Using these equations, the electron density of our MoS_2_ films was determined from the spectral weight of trion PL according to

$\frac{I_{tr}}{I_{total}}=\frac{\frac{\gamma_{tr}}{\gamma_{ex}}\frac{N_{tr}}{N_{ex}}}{1+\frac{\gamma_{tr}}{\gamma_{ex}}\frac{N_{tr}}{N_{ex}}}\approx\frac{{4.4\cdot{10}^{-14}n}_{el}}{{1+4.4\cdot{10}^{-14}n}_{el}}$ (Eqn. S7)

where ${I_{X^{-}}/I}_{total}$ is the trion PL spectral weight. For our analysis, $\gamma_{tr}/\gamma_{ex}$ is ≈ 0.1 from trion and A-exciton PL ratio.

**Figure S8.** Exciton population dynamics for MoS₂ films grown at 900 °C and 1000 °C, obtained by integrating the photoluminescence spectra from 1.75 eV to 1.9 eV. Biexponential fits to the data are shown as solid black lines. The comparison indicates that excitons in the 1000 °C-grown film recombine more rapidly, consistent with enhanced nonradiative pathways arising from heterogeneous substrate-induced charge doping and the associated trion formation.


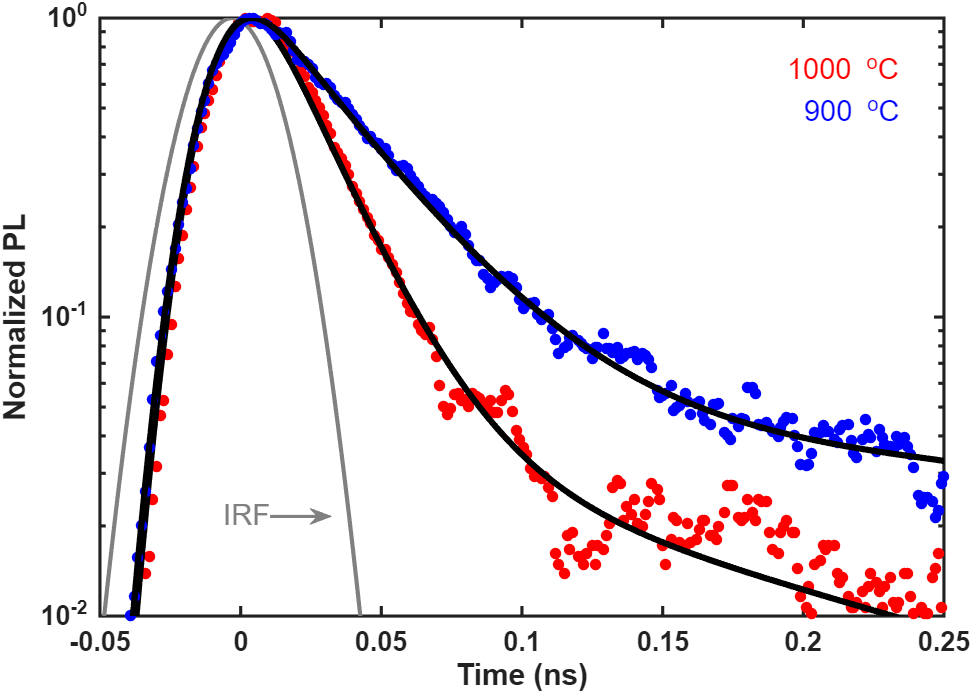


We estimate exciton lifetimes in MoS_2_ films by fitting normalized PL decay traces with a biexponential function, F(t) = a*exp(-t/τ1) + (1-a)*exp(-t/τ2), where (τ1) and (τ2) denote the short and long lifetimes, respectively, and (a) gives their relative contributions to the overall decay. The decay kinetics were convoluted with the instrument response function and optimized using the Unconstrained Nonlinear Nelder-Mead simplex algorithm in the MATLAB Optimization Toolbox. **Table S1** summarizes the extracted lifetimes, amplitudes, and weighted average lifetimes for each film.

**Table S1.** Biexponential Fit Parameters for PL Decay Kinetics in Figure S8

| Sample | a | τ_1_ (ps) | τ_2_ (ps) | τ_ave_ (ps) |
| --- | --- | --- | --- | --- |
| 1000^o^C growth | 0.98 | 26 | 200 | 30 |
| 900^o^C growth | 0.97 | 45 | 700 | 65 |

**Section V:** **Characterization of sapphire substrates after MoS_2_ sample transfer**


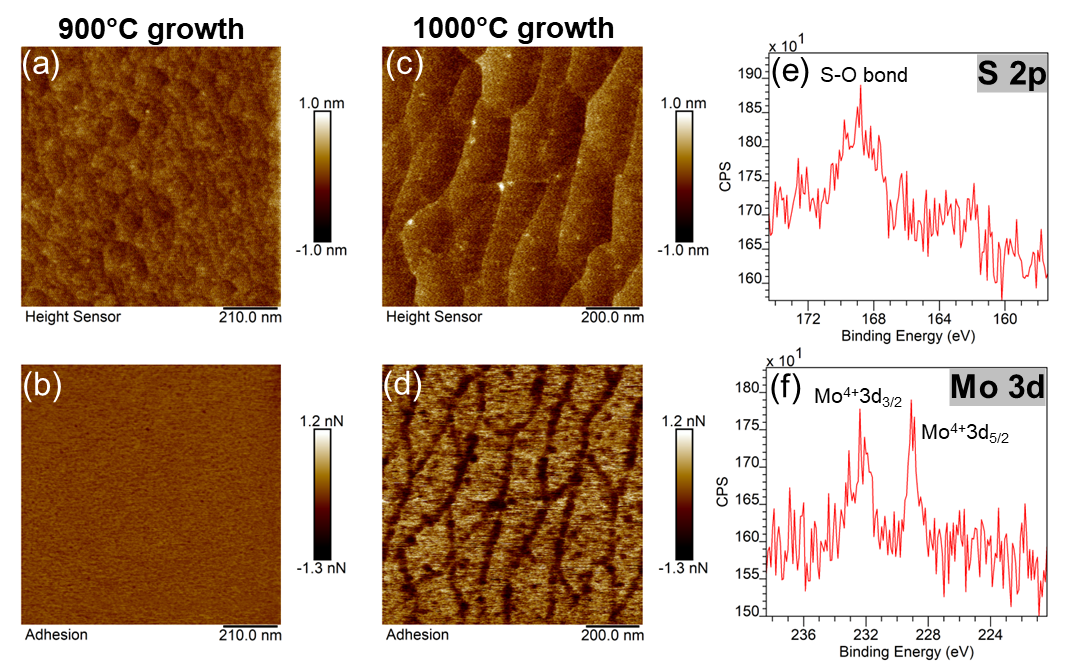


**Figure S9.** Atomic force microscopy (AFM) height and adhesion channels obtained by scanning sapphire substrates after removing MoS_2_ films grown on top of the substrates at (a-b) 900 ^o^C and (c-d) 1000 ^o^C. The contrast observed from the AFM adhesion channel in (d) reveals residue along the step edges of the sapphire substrate after removing the MoS_2_ film grown at 1000 ^o^C. (e,f) X-ray photoelectron spectroscopy of the substrate after transfer shows the presence of Mo and S in the residue. This result suggests that bonding between MoS_2_ and sapphire step-edges makes removing MoS_2_ from these sites challenging.

**Section VI: Kelvin Probe Force Microscopy Measurements:**

In Kelvin probe force microscopy (KPFM), the measured contact potential difference (CPD) between the tip and the sample is given by:

$$V_{CPD}= \frac{\phi_{tip-}\phi_{sample}}{e}$$

where $\phi_{tip}$​ and $\phi_{sample}$​ are the work functions of the tip and sample, respectively, and $e$ is the elementary charge. Since the work function of a semiconductor is given by:

$$\phi_{sample}= \chi+E_{C}-E_{F}$$

where $\chi$ is the electron affinity, $E_{C}$ is the conduction band minimum, and *E*_F_ is the Fermi level, V_CPD_ directly corresponds to local shifts in the Fermi level. Because these local shifts exclusively happen at the step edges of the 1000 ⁰C sample, we attribute these shifts to charge doping effects from the underlying sapphire substrate. Crucially, since the same tip was used throughout the scan for each sample, the differences in CPD between the step edge and terrace regions are independent of the absolute value of $\phi_{tip}.$ As done in the present study, KPFM has been widely used in 2D TMD systems as a tool to resolve relative work-function variations associated with nanoscale structural features such as grain boundaries^[7]^ and underlying substrate step edges.^[8]^

**Section VII: Channel Length effects of BGFETs fabricated on MOCVD-grown monolayer MoS_2_.**


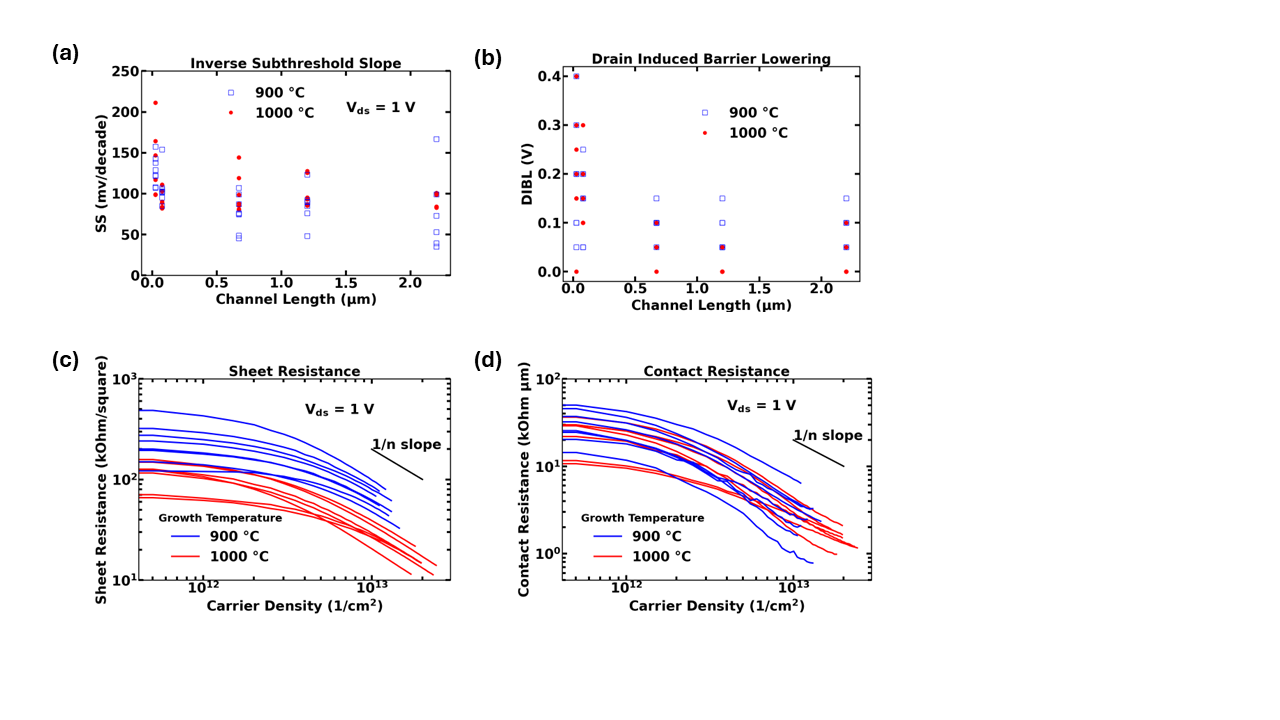


**Figure S10.** (a) Inverse subthreshold slope vs channel length for 900 °C and 1000 °C films showing short channel degradation. (b) DIBL vs channel length for 900 °C and 1000 °C films showing short channel degradation. (c) Sheet resistance and (d) contact resistance vs carrier density for 900 °C and 1000 °C films, on a loglog scale, with expected 1/n slope shown in black.

**Section VIII. References**

[1] M. Liu, J. Liao, Y. Liu, L. Li, R. Wen, T. Hou, R. Ji, K. Wang, Z. Xing, D. Zheng, J. Yuan, F. Hu, Y. Tian, X. Wang, Y. Zhang, A. Bachmatiuk, M. H. Rümmeli, R. Zuo, Y. Hao, *Adv Funct Mater* 2023, *33*, 2212773.

[2] A. Michail, N. Delikoukos, J. Parthenios, C. Galiotis, K. Papagelis, *Appl Phys Lett* 2016, *108*, 173102.

[3] W. H. Chae, J. D. Cain, E. D. Hanson, A. A. Murthy, V. P. Dravid, *Appl Phys Lett* 2017, *111*, 143106.

[4] V. E. Kudryashov, S. S. Mamakin, A. É. Yunovich, *Tech Phys Lett* 1999, *25*, 536.

[5] S. Mouri, Y. Miyauchi, K. Matsuda, *Nano Lett* 2013, *13*, 5944.

[6] H. Shi, R. Yan, S. Bertolazzi, J. Brivio, B. Gao, A. Kis, D. Jena, H. G. Xing, L. Huang, *ACS Nano* 2013, *7*, 1072.

[7] D. Moore, K. Jo, C. Nguyen, J. Lou, C. Muratore, D. Jariwala, N. R. Glavin, *npj 2D Materials and Applications 2020 4:1* **2020**, *4*, 44.

[8] Y.-C. C. Lin, B. Jariwala, B. M. Bersch, K. Xu, Y. Nie, B. Wang, S. M. Eichfeld, X. Zhang, T. H. Choudhury, Y. Pan, R. Addou, C. M. Smyth, J. Li, K. Zhang, M. A. Haque, S. Fölsch, R. M. Feenstra, R. M. Wallace, K. Cho, S. K. Fullerton-Shirey, J. M. Redwing, J. A. Robinson, *ACS Nano* **2018**, *12*, 965.
